# Exploring challenges and mitigation strategies towards practicing Teledentistry

**DOI:** 10.1186/s12903-022-02685-2

**Published:** 2022-12-30

**Authors:** Ayesha Fahim, Zakia Saleem, Khizar Ansar Malik, Komal Atta, Rizwan Mahmood, Mohammad Khursheed Alam, Ahsan Sethi

**Affiliations:** 1 University College of Dentistry, University of Lahore, Lahore, Pakistan; 2 University Medical and Dental College, University of Faisalabad, Faisalabad, Pakistan; 3 Azra Naheed Dental College, Lahore, Pakistan; 4 College of Dentistry, Jouf University, Sakaka, Saudi Arabia; 5 QU Health, Qatar University, Doha, Qatar; 6 University College of Medicine, University of Lahore, Lahore, Pakistan

**Keywords:** Challenges, Solutions, Teledentistry, Telehealth, Virtual patient, Virtual dentistry

## Abstract

**Background:**

Since the beginning of the COVID-19 pandemic, many dentists have opted for Teledentistry as a mechanism for patient consultation, oral lesion evaluation, diagnosis, and monitoring. The current study explores the challenges faced and potential solutions proposed by dentists practicing Teledentistry in a developing country like Pakistan.

**Methods:**

A qualitative case study was carried out from January to December 2021. A purposive maximum variation sample of 10 dentists was interviewed in two focus groups. The interview guide was developed using the technology–organization–environment framework. The data was transcribed verbatim using otter.ai. The analysis involved immersion in the data and open coding. The conceptually related codes were synthesized into themes and subthemes.

**Findings:**

The study found various Personnel, Technological and Organizational challenges, and potential solutions from those practicing Teledentistry. The challenges included operational cost, minimal financial returns, lack of awareness, hardware and software support, and other challenges related to the availability of specialization, accessibility, and institutional encouragement. They suggested Institutional Based Practice, staff training, hiring, development of government regulations, and supporting infrastructures such as designated space, central registry, internet, and using/building software to provide 3D images as solutions.

**Conclusion:**

Teledentists face Personnel, Technological and Organizational challenges and related potential solutions from those practicing Teledentistry in Pakistan. Government should encourage Teledentistry to reduce long-term costs, encourage preventive services and enable rural access to dental care. They should also involve all stakeholders to develop regulations for practicing Teledentistry in Pakistan.

**Supplementary Information:**

The online version contains supplementary material available at 10.1186/s12903-022-02685-2.

## Background

In March 2020, the World Health Organization (WHO) declared coronavirus disease (COVID-19) a pandemic. It was a public health emergency that sparked worldwide concern [1]. Most of the hospitals were designated for screening and management of COVID-19 cases. To avoid the risk of cross-infection, all elective services in the hospitals and private clinics including those related to Dentistry were suspended [2].

Given the unique nature of the dental practice, the risk of cross-infection is always a concern. Exposure to blood, body fluids, saliva, and airborne pathogens in dental practice may serve as a potential source for transferring COVID-19 [3, 4]. Anecdotal evidence suggests that COVID-19 may become endemic. Teledentistry was introduced in many countries as an innovative solution to minimize the risk of cross-infection and continue dental practice during the current pandemic, as well as beyond. Teledentistry involves diagnosis, consultation, provision of dental care, management, and advice by means of telecommunication and technology [5]. It facilitates dental care, guidance, and patient education remotely and at a safe distance. Teledentists can use instant messaging applications or video calls as a source of communication for consultation [6]. Teleconsultation, telemonitoring, teletriage, and telediagnosis are various subunits of Teledentistry [7]. Teleconsultation proves helpful in the reduction of non-urgent referrals of patients and ultimately reduces the burden on busy healthcare departments. Remote oral health assessment by teletriage prioritizes patients seeking urgent care along with the reduction of travel which proves beneficial for patients with geographical and socioeconomic difficulties [8]. Telediagnosis can also be made by the exchange of patient’s records, radiographic and intraoral images [9].

Coined in 1997 by Cook, Teledentistry (TD) was initially referred to as videoconferencing with patients at a distance [10]. It was first introduced in a United States project to assess the oral health of US army service men [11]. Other developed countries like UK, Australia, and Canada quickly followed and adopted TD with an increase in technological advancements [12]. In America and Europe, TD has expanded exponentially and more than 80% of dentists have a positive perception and acceptance of TD [7]. Some countries e.g., Saudi Arabia and Qatar have included TD in their vision 2030 developmental model [13, 14]. However, many developing countries still lag in the use of technology in clinical dental practice.

As per Pakistan Medical Commission, there are 27,428 registered dental surgeons managing oral health services in all the public and private-sector healthcare facilities for an estimated population of 235 million in the country. This shortage of qualified dental professionals in primary, secondary, and tertiary healthcare facilities, arguably, makes Pakistan most suitable for the establishment of Teledentistry. In May 2005, the World Health Assembly Resolution 58.28 urged countries to develop eHealth strategies in their countries [15, 16]. As a World Health Organization (WHO) member state, Pakistan initiated an eHealth program. Some awareness and advocacy seminars were also conducted, but these did not include dentistry [17, 18]. The third global survey on eHealth conducted by the WHO Global Observatory for eHealth (2015) reported no eHealth-related national policies, strategies or legislation in Pakistan [19]. Their survey also reported a lack of requisite skills and e-learning programs for students in dentistry, nursing, and pharmacy. Our literature search only identified one KAP study on Teledentistry before the pandemic from the capital city (most advanced) in Pakistan. The participants in that study did report having some knowledge of Teledentistry. However, only 9/155 participants answered about the software correctly. Moreover, all the participants mentioned that they had never practiced Tele-dentistry anywhere in Pakistan [17]. The Pakistan National Health Vision 2025 aims to improve access to healthcare using Information and Communication Technologies (ICT). This opens up opportunities for developing Teledentistry in the country, which was adopted as an emergency response to the COVID-19 pandemic in 2020 [2, 20, 21]. Some short courses to train healthcare professionals in Teledentistry software were also conducted by various institutions. This resulted in its uptake by several dentists in Pakistan. In Pakistan, most of the dentists work for public/private dental institutions in the morning and run private clinics (self-employed) in the evening. They utilized Teledentistry for the prevention and early detection of carious lesions and soft tissue lesions. It was also used for provisional diagnosis and treatment planning of difficult cases by easy access to various specialists through transfer of radiologic images [22–24].

A large number of studies have quantitatively assessed the knowledge and perception of dentists regarding TD [25, 26]. Some studies suggest the incorporation of Teledentistry into routine dental practice as the need of the hour [5, 10, 27, 28]. These studies also indicate that 60–70% of dentists had never practiced Teledentistry before the onset of COVID-19 [2, 25]. Despite positive perception, 37% of the dentists think practicing Teledentistry is challenging [2]. Although important, the studies about knowledge and awareness are not sufficient for the smooth implementation of TD [21, 29]. To achieve the desired results of these visions in oral health care, there is a need to explore challenges faced by those currently practicing Teledentistry qualitatively. However, research in Teledentistry is lagging when compared to the medical fraternity and most of the Teledentistry studies are from developed European countries, leaving a wide gap in knowledge on the issues of the Asian and developing countries. Insight into the challenges can facilitate implementation of Teledentistry both in the undergraduate/postgraduate curriculum as well as the practices. All previous studies have quantitatively assessed the perception and awareness of dentists via close-ended questionnaires. The current study qualitatively explores the challenges faced and potential solutions proposed by dentists practicing Teledentistry in Punjab, Pakistan, using the technology–organization–environment (TOE) framework.

## Methods

Using an interpretivist approach, a qualitative case study was carried out from January to December 2021. The case study allowed for an in-depth exploration of complex and multi-faceted challenges experienced by the dentists practicing Teledentistry during the COVID-19 pandemic era [30]. Ethical approval was provided by the Ethical Review Board of Azra Naheed Dental College (ANDC/RAC/34/06).

A purposive maximum variation sample of 10 dentists was interviewed. Teledentists with diversity in job experience, professional credentials, geographical distribution (Punjab, Pakistan), and workplace (private/public-sector) were invited to participate in the study through email and telephone. A participants’ information sheet with a brief description of the study, objectives, methodology, and expectations along with a consent form was shared through email with 30 dentists. After two reminders, we received written informed consent from 15 Teledentists. However, only 10 Teledentists attended the two focus group interviews (n = 5 each). Focus group interviews have been extensively used in qualitative research to gain in-depth knowledge, and perspectives and attitudes of people about a certain topic [31]. We preferred using Focus group interviews over individual interviews as it is cost-effective, and encourages participant interactions which result in increased spontaneity and greater input [32]. Also, most of the participants knew each other and felt more comfortable having the interview together. The procedure for FGD was undertaken following the AMEE guide (no. 91) on ‘Using focus groups in medical education research’ [33].

To increase the comprehensiveness of the data collected, and to make the data collection more efficient, an interview guide (Additional file 1) was developed using the technology–organization–environment (TOE) framework that explains the technology/innovation adoption [34]. It is an organization-level theory that explains decisions regarding the adoption of technological innovations based on technological, organizational, and the environmental contexts. The technological context focuses on the technologies, their perceived usefulness, technical complexity, and organizational compatibility. The organizational context refers to the institutional characteristics, support, specialization available, and other resources. The environmental context refers to governmental encouragement, supporting infrastructures, sociocultural issues, and other operational enablers and inhibitors [35]. The interview guide explored challenges and solutions based on these contexts. The guide was also shared with qualitative research and healthcare technology experts (n = 5) for validation. The questions were sequenced and rephrased in accordance with the expert review and piloted (n = 2) to ensure comprehensiveness and clarity. Since the interview guide was semi-structured, it allowed for prompts and additional questions for an in-depth exploration from the participants.

Focus group interviews were conducted online via Zoom in September 2021. One of the researchers (AF) acted as the moderator of the session. The second researcher (KAM) acted as a non-directive moderator to gain spontaneity and depth of information [36]. To minimize researcher influence and bias over the discussion, a third researcher (ZS) was present as a note-taker to note down the main points and non-verbal cues [31]. The entire session took ninety minutes to complete. The focus group interviews were spaced two weeks apart to allow for an iterative approach to data collection and analysis. This helped the researchers explore areas in-depth and claim saturation when new ideas or responses stopped coming.

### Data analysis

The data were transcribed verbatim using ‘Otter.ai’, which converted speech into written quotes [37]. The transcripts were reviewed to ensure accuracy. The review process also helped with familiarization with the data [38]. Three authors (AS, AF, ZS) immersed themselves in the entire data and developed the first set of coding framework [39]. The transcript was read and re-read and memos were written to record the thought processes. The three set of coding frameworks were then discussed. The generating initial codes organised data inductively based on similarity, resulting in a list of non-overlapping challenges faced by teledentists [40]. Some codes were in-vivo, while others were influenced by the theoretical framework. This initial coding framework was used for indexing. The conceptually related codes were compared, connected and synthesized into subthemes and themes [39]. The sub-themes and themes were continuously refined with constant deliberation among the researchers. For member checking, the transcripts and themes were shared with the participants through email and they were asked to ascertain if the analysis represented their perceptions and experiences.

## Results

Ten teledentists with a range of experience in teledentistry from both public and private-sector participated in the study. The participants were at different stages of their careers (Table 1).

**Table 1:** Participant characteristics

Characteristics	Frequency	Percentage
*Gender*		
Male	4	40
Female	6	60
*Age group*		
26–35 years	5	50
36–45 years	3	30
46–55 years	1	10
56 and above	1	10
*Sector*		
Public	6	60
Private	4	40
*Years of graduation*		
Before 2000	1	10
2000 and after	9	90
*Academic qualification*		
BDS	2	20
Postgraduate trainee (basic sciences)	1	10
Postgraduate trainee (clinical sciences)	4	40
Consultants	3	30
*Years of experience in Teledentistry*		
0–2 years	6	60
3–5 years	3	30
> 5 years	1	10

The various challenges and potential solutions faced by those practicing Teledentistry were identified and grouped into three broader themes: Personnel (focusses on the individuals), Technological (focusses on the technology) and Organizational (focusses on institutional support and resources) factors.

### Personnel factors

Among personnel factors, the participants reported operational cost, minimal financial returns, lack of awareness and acceptability amongst patients as challenges. All of the participants were practicing Teledentistry in a teaching hospital/ institution. None of them were able to manage it independently in their private clinics. Setting up Teledentistry in a private clinic is an expensive option, as they may have to bear its cost themselves, with no/minimal financial returns for the investment. They suggested that it is only practical as part of Institutional Based Practice.

*We cannot practice Teledentistry independently. We rely on institutional resources.*

Some participants mentioned that the patients were not aware of the process of online consultation, which led to them being unsatisfied. Even the patients needed to have some basic understanding of the requirements and the process.

*I asked my patient to send me intraoral images. It took me 2 hours to explain my patient how to take clear pictures and at what angle.*

There is also lack of acceptability from the patients as they felt the lack of clinical touch from the dentist. The patients doubted their diagnosis as they felt that Teledentists didn’t examine them physically.

They suggested a need for massive campaigns to raise awareness in order to make it more feasible and acceptable for the patients.

*I think that advertisement by the government* … *was not that good. People were not given awareness to the events through the media or through the television or to billboards or sign boards or any pamphlets or any kind of campaigns. No campaign was launched.*

### Technological factors

The participants reported hardware and software support, specialization available, accessibility and other technological limitations as challenges. They mentioned lack of formal training in the use of softwares and other aspects of Teledentistry. They suggested a need to conduct workshops and trainings.

*We were given a demo by the people that came from ... health department. Only one demo regarding the working but it was not very well told it was just a simple briefing that how you have to login and what is your login.*

Some even mentioned that it should be considered for inclusion in the formal curriculum at the undergraduate and postgraduate level. They also suggested forming a social network community for Teledentists where they can discuss the challenges and learn from others’ way to resolve them.

*If Telemedicine and Teledentistry is the future of medicine and dentistry, we should introduce a curriculum or course on it. IT applications, ethical values, software use etc. should be taught in that curriculum or course.*

The staff working in clinical departments do not qualify to support Teledentists during online sessions. Support staff well versed with the technology is required in both places, the institution as well as patient center.

*The helper at the patient center did not know how to take intraoral pictures. I asked him to show me the carious lesion on video call but he could not handle camera.*

The accessibility to the computer, software and internet to the patient in rural areas was also a challenge. Most of the patients were uneducated and they found the process of connecting to a doctor for consultation as complex and exhausting. The internet connectivity was also a barrier. Improper communication among the patient and dentist, can lead to frustration and misdiagnosis. The participants suggested provision of centres in rural areas with software, staff and good bandwidth internet for the patients, so that they can connect to a Teledentist comfortably.

*And most of the patients we looked at, did not even have a good WiFi signal. So, most of the calls were dropped between the treatment that we were telling them.*

They also reported technological limitations, for instance they are only able to see a two-dimensional picture on the video call due to which they sometimes felt clueless while making a clinical diagnosis. They suggested using/developing hardware and software that can record, store, and project patient 3D images. Artificial Intelligence can also be used to help complement the diagnosis and management.

*How can I look at the clinical findings and the radiographic findings only by calling him through the video chat?*

### Organizational factors

Among organizational factors, the participants reported a lack of institutional encouragement, designated roles & responsibilities, government regulations and supporting infrastructures like central registry of dental records as challenges towards sustainability of Teledentistry in Pakistan. There were no formal department and designated staff for Teledentistry at institutional level. The institutions were reluctant to hire staff or provide additional renumerations.

*I am practicing in a private hospital, and it is difficult for our administration to bear the support staff pays, and at times they are not happy with this.*

The institutions had limited encouragement for the operational difficulties with Teledentistry and the dentists were balancing multiples responsibilities. They were juggling their primary clinical department workload along with the Teledentistry.

*Most of the time when I used to go upstairs for Teledentistry, there was a big queue of patients waiting for me to get checked*

They suggested hiring new or redesignating existing staff with protected time for Teledentistry to make it more efficient and effective.

*A department of Teledentistry should be established which includes trained faculty members (or general dentists) solely for this purpose, trained IT and support staff, 24 h electricity and internet connection provided to Teledentists and remote areas where we wish to start Teledentistry.*

The participants were also concerned about lack of guidelines and regulations on Teledentistry in the country e.g., on issues related to patient confidentiality, consent, data distribution, data safety, consultation, validity of diagnosis, referral and other legal liabilities. In the absence of regulations, every dentist was applying their own ideas of consultation, management plan and referral processes. They suggested developing clear regulations and guidelines related to Teledentistry by the health professions regulatory body in the country.

*If some patient showed me his/her pictures during a video-based session, I am not sure how the system will ensure the confidentiality of patient and it is bothering me.*

They also reported insufficient infrastructure for practicing TD in the country. The was no central register with digital oral records for the patients to store and compare with, compromising the clinical diagnosis further. They opined the need for governmental support, development of infrastructure and appropriate budget allocation to make Teledentistry more sustainable.

*Government needs to provide some basic infrastructure to practice and promote Teledentistry.*

## Discussion

Teledentistry is an innovative method of health service delivery that can assist dentists in providing online consultation, suggesting investigations, follow-ups and referrals. To the best of our knowledge, this is the first study that qualitatively explore challenges and barriers of dentists practicing TD. This study also attempted to seek out practical solutions to challenges in teledentists’ viewpoint. Our study identified three key factors associated with teledentistry practice in Pakistan: personnel factors, technological factors and organizational factors.

For our study, we could not find dentists practicing TD in their private dental clinics. All participants were working under the umbrella of some private or government institution. A reason for this might be that the equipment and set-up required for TD is expensive. Moreover, it does not only require stable internet connection from the dentist, but the patient as well [41]. Literature reveals that patients generally prefer visiting dental clinics instead of TD [42], but in pandemic conditions several institutions had setup patient centers in remote areas to facilitate dental patients for free or on minimal charges. The establishment of this setup is high-priced and considered an emergency service during the Pandemic. Thus, we believe that dentists prefer to utilize organizational facilities instead. Moreover, many patients do not have the means to access the internet or have an internet-enabled device or a reliable internet connection. All dentists in Pakistan did not receive a formal training in TD but they still managed to initiate TD practice. Without formal trainings and certifications, TD might be disastrous with negative effects [43]. Where TD was introduced to reduce quackery in remote areas, untrained teledentists might not be able to provide full support to dental patients. Dentists who managed to obtain formal online trainings in TD, felt overwhelmed by the lack of software and technical support, lack of trained supporting staff and patient awareness [44].

Patient acceptance was another major concern of teledentists. Patients were generally dissatisfied with online consultancy and felt uncomfortable describing their symptoms. Recent literature has indicated that patients preferred a stay-at-home consult with physicians and dentists during COVID-19 to avoid contamination [7, 45], but our study depicts otherwise. The reason suggested for this contrast is that patients in Pakistan especially in rural areas are not well-versed with online communication. An uncritical embrace of technology should not ignore the Rural-Urban divide and economic disparity among different societal groups in many in countries such as Pakistan. Access to internet and technology is still a luxury. Besides poor-quality networks, most of the population also has a low level of digital literacy and affordability. In some regions, there is no electricity, no telecommunication or even a telephone, while in other regions, internet access has been purposefully restricted for security reasons. Moving for internet access may put public at a greater risk from the pandemic. Moreover, the context of pandemic was characterized by large level of threat, including fear from technology [46] which caused a generalized apprehension amongst public. Teledentists suggested a need for massive campaigns to acclimatize the public and raise acceptability level. Literature has suggested public awareness campaigns in the form of single-exposure short media, gate keeper programs and long programs involving repeated exposure [47] that could be useful to promote Teledentistry in Pakistan.

Another major factor explored through this study was the ‘technological factors’. According to teledentists, lack of formal training and poor internet connection for both the dentist and the patient greatly hinders the TD process. Studies have shown that organizations who have forced TD in COVID without proper preparation and software/internet management have failed in its implementation [48]. Literature dictates that lack of formal trainings leads to mismanagement and enhances patient apprehension [49]. Some private organizations hesitate to spend extra amount on equipment and technical support, where this happens dentists might lose motivation towards TD [50]. The East Surrey Hospital has launched telephonic consultation in their department to facilitate patient-dentist communication [47]. Institutes in Pakistan wanting to establish a ‘Department of Teledentistry” might perform better if they provide multiple options for patients including telephonic, video-conferencing, virtual reality and augmented reality under the same roof. Similar centers could be established for patients especially in rural areas to provide ease and accessibility. According to our participants, establishment of a formal TD department is a necessity to optimize TD in the region. This notion has been emphasized in several previous studies [51] as well. University of Rochester in USA presume that a formal department not only regulates the functioning of Teledentists, it provides a practical and cost-effective way to improve oral healthcare in rural and disadvantaged children [51]. A TD network was established in California USA, initiating license of teledentists, which helped regularize TD in California [52]. Owing to the limited funds and bleak technological advancement in Pakistan, the governing bodies might not be able to mirror the establishments of USA, but these examples could be used as foundation for implementation in our context. The current study also reported limitations of TD, e.g., the dentists are only able to see a two-dimensional picture on the video call, which makes the diagnosis difficult. The literature also reported concerns related to confidentiality of patient information and improper diagnosis and/or management [22]. Therefore, the patient should be informed about the inherent risk involved as part of the standard informed consent.

Our study participants considered lack of institutional encouragement, undefined pupil roles and improper infrastructure as barriers to TD compiled as ‘organizational factors’. Like most departments in developing countries, specialist teledentist are not usually hired. Existing faculty members are given additional tasks without any clear incentives [53]. Multiple tasks lead to burnout and stress of individuals. This might be the cause of this barrier. In Pakistan, specific guidelines are not yet available for TD. USA and Australia has launched its guidelines, so has other well developed countries [54], but universal guidelines from WHO or other statuary bodies is not available yet. This might lead to a generic confusion and disagreement amongst practicing teledentists. The ‘British General Dental Council has provided key principles for teleconsultations. It identifies the responsibilities of dentists and provides a safeguard for patients engaged in teleconsultation [55]. Without such guidelines and principles, TD cannot be monitored. This might be the reason our participants suggested that proper guidelines must be available especially for sensitive matters like patient consent, data confidentiality, legal issues etc. With appropriate support from government and TD specialists, our participants believe that TD can be established and regularized in Pakistan.

A recent study in Nepal revealed that although dentists were familiar with the terminology, they had never practiced it before covid pandemic [56]. Similar observations were available in studies conducted in Saudi Arabia and Iran where 75% dentists were not aware of Teledentistry and in India, 98% of dentists were unfamiliar with teledentistry before the pandemic [57–59]. They have struggled with the medicolegal issues concerned with Teledentistry practice especially in rural areas [60, 61]. A whooping 70–80% majority of Malaysian dentists expressed uncertainty in the usefulness of Teledentistry in their country [62]. A nationwide study conducted in Brazil confirmed that their dentists were not adequately prepared to implement Teledentistry [63]. A global survey predicted the potential use of Teledentistry worldwide but to avail its optimum benefits for developing countries, in-depth research is required about their business models, guidelines and policies and support from all stakeholders including government, institutions and dentists [64]. So, most of the developing countries have not fully implemented tele dentistry, which is in line with the findings in our study. Therefore, we believe that our findings are transferable to other similar contexts, who adopted teledentistry during the covid19 pandemic and still working towards developing it further to serve the needs of remote and rural areas in the country. It is also pertinent to mention that all the studies above were quantitative in nature while our study was qualitative, exploring the challenges and enabler towards teledentistry in-depth.

Our study has a few limitations. We only interviewed Teledentists from Punjab region of Pakistan, which is the largest, most populous and urbanized Pakistani province. For a comprehensive picture of challenges, the future studies should identify challenges from any dentists using TD in private clinics and other stakeholders like patients and policy makers. Moreover, all the dentists in our study were practicing Teledentistry in a teaching hospital/institution. We could not find any dentists practicing it solely in their private practice. Moreover, the authors are not practicing dentists but working in academia and this may have affected their interpretations of the data. We did not specifically ask or probe the participants about any illegal acts because unlike USA, where it was illegal for a dentist in one state to advice patients from another state till 2002, there are no such obligations in Pakistan. Moreover, the participants did not mention committing any illegal acts in their teledentistry practice. Either they may have deliberately avoided the discussion, or they were not aware due to unavailability of regulations from the government regarding patient consent process, management of patient information and potential for malpractice in Teledentistry, as in other countries [65].

## Conclusion

This study provides an in-depth understanding of the challenges and potential solutions from those practicing Teledentistry in Pakistan. Teledentists are facing several Personnel, Technological and Organizational challenges. The challenges included operational cost, lack of patient awareness, unavailability of specialization, inaccessibility and lack of institutional encouragement. Institutional Based Practice, training, government regulations and supporting infrastructures such designated space, central registry, internet may help make Teledentistry sustainable. Government should encourage Teledentistry to reduce long-term costs, encourage preventive services and enable rural access to dental care. They should also involve all stakeholders to develop regulations for practicing Teledentistry in Pakistan.

## Supplementary Information

**Additional file 1.** Interview Guide.

## Data Availability

The datasets generated and analyzed during the current study are not publicly available because participants of the study did not agree for their data to be shared but are available from the first author on reasonable request.
