# Supplementary material for: Exploring challenges and mitigation strategies towards practicing Teledentistry
Source: BMC Oral Health. 2022 Dec 30;22:658. doi: 10.1186/s12903-022-02685-2 (PMC9803256; doi:10.1186/s12903-022-02685-2)
Supplement: Supplementary file 1 — Additional file 1. Interview Guide. [file 12903_2022_2685_MOESM1_ESM.pdf]

Interview Guide

Exploring challenges and mitigation strategies towards practicing Teledentistry: A qualitative study

Thank you for participating in this study, I acknowledge how busy you are and really appreciate the time you have taken.

Through this interview; I am interested in finding out about your tele-dentistry practice and any challenges and mitigation strategies towards it. You can respond to the questions with comments, thoughts and stories of your own.

As mentioned in the participants' information sheet, this interview will take about 30 minutes and it will be audio recorded. I assure you that any information you share will be kept confidential and you will not be identified in any future reports and publications. Please do not hesitate to ask any questions you may have.

So, if you are ready, shall we begin the interview?

Can you briefly share your experience of practicing Teledentistry?

Have you practiced Teledentistry privately or as part of an institute/organization? Explain the reason for your preference?

How teledentistry is useful in our context?

What are some of the enablers towards using Teledentistry in Pakistan?

What are some of the challenges have you faced while practicing Teledentistry in Pakistan?

Did you find any difference in in-person and teledentistry practice? Explain in detail.

How has your organization contributed to facilitation of Teledentistry practice?

Did the government play any role in facilitation and awareness of Teledentistry? What was it?

How have your patients reacted to the Teledentistry practice?

How have you altered your practice from in-person to Teledentistry?

How has technology affected your practice? Positive or negative, explain.

How can teledentistry improve in Pakistan? Explain.

Is there anything else you would like to add before I conclude?

Thank you.
